# Perceptions of satisfaction, usability and desirability of the DEKA Arm before and after a trial of home use

**DOI:** 10.1371/journal.pone.0178640

**Published:** 2017-06-02

**Authors:** Linda J. Resnik, Matthew L. Borgia, Frantzy Acluche

**Affiliations:** 1 Research Department, Providence VA Medical Center, Providence, Rhode Island, United States of America; 2 Health Services, Policy and Practice, Brown University, Providence, Rhode Island, United States of America; University of Colorado Boulder, UNITED STATES

## Abstract

**Objectives:**

To: 1) describe perceptions of satisfaction with and usability of the DEKA Arm and preferences for the DEKA Arm or personal prosthesis; 2) compare perceptions of satisfaction and usability by DEKA Arm configuration level; and 3) evaluate satisfaction and usability for study completers and non-completers; and for those who did and did not want to receive a DEKA Arm.

**Methods:**

The study had 2 phases: in-laboratory (Part A) and home trial (Part B). 32 participants with amputation, (50% transradial, 38% transhumeral and 13% shoulder) completed Part A and 18 completed Part B 16 (89%) of whom were prosthesis users at baseline. Measures of satisfaction, usability and user preferences were administered. Responses were compared for completers of Part A only and completers of Parts A and B. Preferences for the DEKA Arm over personal prosthesis and proportion of participants who wanted to receive a DEKA Arm were evaluated. Relationships between satisfaction, usability and desire to receive a DEKA Arm were examined.

**Results:**

At end of Part A, 22 (69%) of the 32 participants who completed in-laboratory training wanted to receive a DEKA Arm and 5 (16%) might want one. At end of Part B, 14 (88%) of 16 prosthesis users who completed the home trial preferred the overall function of the DEKA Arm, 13 (81%) preferred DEKA hand function and 14 (88%) preferred DEKA wrist function to their own prosthesis. In contrast, 14 (88%) preferred the weight and 13 (81%) preferred the look of their own prosthesis. Most aspects of the DEKA Arm were rated “easy” to use. No items were rated as “difficult”. Users were satisfied with most aspects of the DEKA Arm, except for the weight, shoulder appearance and harnessing. There were few differences in perceived usability or satisfaction by configuration level. Findings about desire to receive a DEKA Arm pertain only to study completers. Non-completers viewed the DEKA Arm less favorably than completers. Satisfaction was strongly related to participants’ expressed desire to receive a DEKA Arm in the future.

**Significance:**

To maximize likelihood of adoption of the DEKA Arm, findings suggest that both an in-laboratory and a home use trial may be useful prior to finalizing a recommendation for prescription.

## Introduction

Persons with upper limb amputation are more likely to go without using a prosthesis as compared to persons with lower limb amputation.[1] A review of 25 years of research found that mean prosthesis abandonment rates amongst adults with upper limb amputation were 26% for body-powered device users and 23% for myoelectric device users. [2] Upper limb prosthesis use and abandonment have been studied extensively, [1, 2, 3, 4, 5, 6–15] and many factors associated with increased likelihood of device abandonment or rejection have been identified including: proximal level limb loss, female gender, perceived lack of need for a device, problems with prosthesis reliability [15] and cosmesis, inadequate prosthetic training, delay in prescription of a device after amputation, dissatisfaction with functionality/usefulness of the prosthesis, and discomfort from wearing the prosthesis or socket.

Although some factors reported to be associated with device abandonment, such as level of limb loss and gender, are set, [3] other factors, such as the functionality of the prosthesis might be addressed by technological advances and better equipment. In fact, there is some evidence that prosthesis abandonment rates have declined over time, at least amongst Veterans with amputations, likely due to advances in care and technology.[12] Nevertheless, even in this group, complete prosthesis abandonment was reported in 22% of unilateral combat amputees from Operation Enduring Freedom (OEF) and Operation Iraqi Freedom (OIF), many of whom received timely prosthetic prescription and training within specialized amputation centers and were provided with multiple prostheses, including commercially available state of the art devices, as part of usual care.

Intensive research activities over the past decade to advance upper limb prosthetic technology may ultimately contribute to reducing abandonment, but in our view, only if users are satisfied with the resulting new products, and if the perceived trade-offs between weight, speed, complexity and maintenance are considered worthwhile. [14] The DEKA Arm is an example of a new advanced upper limb prosthetic device, approved by U.S. Food and Drug Administration device in 2014, and developed under the Revolutionizing Prosthetics Program through DARPA.[16] The DEKA Arm promises increased functionality with six different powered hand grip patterns, powered wrist movements, simultaneous joint control, and specialized, customizable control schemes. Prior research conducted during the VA Study to Optimize the DEKA Arm compared usability and satisfaction ratings for two prototypes of the DEKA Arm, the Gen 2 and the Gen 3 prototypes, and identified features of the Gen 3 device that would benefit from further optimization including: the weight, the external cables and wires, the hand covering and fingernails. [17]

Although we hypothesize that the user experience of using a prosthesis in a supervised clinical (laboratory) setting is different than the experience of using the prosthesis in a less structured and more natural home environment, little research on this topic has been reported. Thus, it is unclear if or how user perceptions change as a result. One prior study found that upper limb amputees reported continued difficulties with integrating their prosthesis into daily activities, even after extensive prosthetic training. [14]

The VA Home Study of the DEKA Arm (Home Study) was a longitudinal study that involved in-laboratory training to use the Gen 3 DEKA Arm followed by a trial period of home use of the device. Analyses of data from the Home Study reported an overall 57% attrition rate, with 43% of subjects who began the study, completing all activities. [18] Most subject attrition occurred after completion of the laboratory portion of the study, with some subjects declining to participate in the home use portion of the study and others withdrawing during the home trial. [18] Reasons for and predictors of this study attrition are important to understand because withdrawal from the Home study can be viewed as an indicator of unwillingness to adopt the DEKA Arm and thus a proxy for device abandonment. [19] Although qualitative analyses found that 67% of subjects withdrew from the Home Study for at least one reason associated with the DEKA Arm, to date satisfaction and usability ratings of the DEKA Arm during the Home Study have not been quantified. Earlier analyses found that participants in the Home Study who were prosthesis users at baseline were more than 5 times as likely to complete study participation compared to non-users and those with a history of musculoskeletal problems, were 0.2 times as likely to complete study participation. Although bivariate comparisons suggested that amputation level was associated with study completion, this observation did not persist once baseline prosthesis use was accounted for in a multivariate logistic regression model. [19]

Given the recent FDA approval, the expected commercial release of the DEKA Arm, the anticipated high costs of the device, and the need for a skilled rehabilitation team to fit and train patients to use the device,[20] additional research on perceptions of usability and satisfaction of the DEKA Arm are needed. Although the VA Study to Optimize the DEKA Arm reported no differences in user ratings of satisfaction and usability by DEKA Arm configuration level, [17] because of the known relationship between level of limb loss and prosthesis abandonment, additional research to compare perceptions by level of DEKA Arm configuration are warranted. Furthermore, studies are needed to evaluate the relationship between perceptions about device characteristics, users’ interest in using the device at home and receiving a DEKA Arm in the future. Therefore, the purposes of this paper are to 1) describe participants’ perceptions of satisfaction with and usability of the DEKA Arm and their preferences for aspects of the DEKA Arm as compared to personal prosthesis; 2) compare user perceptions of satisfaction and usability by level of DEKA Arm configuration; and 3) evaluate satisfaction and usability for participants who completed the study and those who withdrew; and for those who expressed a desire to receive a DEKA Arm and those who did not.

## Methods

### Gen 3 DEKA Arm

Three configurations of the Gen 3 DEKA Arm are available. The shoulder configuration (SC) which is appropriate for persons with shoulder disarticulation, forequarter amputation or persons with very short transhumeral (TH) amputation; the humeral configuration(HC) which is designed for persons with TH amputation, and the radial configuration (RC) which is designed for persons with transradial (TR) amputation. [16] All configuration levels have six powered handgrip patterns, powered wrist flexion/extension and powered pronation/supination. The HC and SC configuration levels allow powered elbow flexion and extension and humeral internal and external rotation. The SC allows powered shoulder movement in two degrees of freedom (flexion/ extension and abduction/adduction). Control of the proximal joints of SC device is done predominantly through endpoint control, rather than control of individual shoulder and elbow movements. In the DEKA endpoint control scheme the operator controls the endpoint (or terminal device) of the prosthesis in space by moving it up/down, right/left or forward/back. Control of shoulder abduction/adduction movement, called voluntary elbow positioning, is controlled separately from the endpoint control scheme.

The HC and SC DEKA Arms have two modes of operation: hand mode, where controls are used to operate movements of wrist and hand; and arm mode, where controls are used to operate elbow or shoulder movements. The DEKA Arm was designed to be controlled primarily by inertial measurement unit [IMU] foot controls, placed on the top of the feet. [21] However, this primary control method can be supplemented by pneumatic bladders, linear transducers and surface electromyography [EMG] controls. All configuration levels have a standby feature which deactivates all device functions when the prosthesis is powered on as well as a wrist display unit. The wrist display contains LED lights that indicate the power and mode status as well as the grip that is currently selected. All configurations have a manual release button located on the back of the DEKA hand, which, when pressed, releases the mechanical brakes, and thus can be used to reposition the prosthesis, if needed.

The HC and SC DEKA Arms contain an internal battery, but also can be connected to an external battery source for increased battery life. Whereas, the RC devices must be used with an external battery. External batteries are typically worn in a holster, suspended from a waist belt and a battery cable attaches the battery to the prosthesis itself.

### Study design

Data analyzed in this study was collected from participants enrolled in the Home Study, a multisite study involving 3 data collection sites. The study was approved by the Institutional Review Boards of the Providence VA Medical Center, the VA NY HHS, the James A. Haley VA, and the Center for the Intrepid at Brooke Army Medical Center. The Home Study had 2 phases: in-laboratory (Part A) and a home trial (Part B). During in-lab training, all participants were fit with and trained to use the DEKA Arm. In-lab training incorporated use of a virtual reality training environment (VRE) that provided participants with an opportunity to acclimate to features of the DEKA Arm while operating an avatar on a computer screen. Description of the VRE system used in the Home Study is provided elsewhere [16, 22, 23]. At the conclusion of Part A, a battery of self-report measures assessing satisfaction and usability of the DEKA Arm (see below) were completed. Eligible participants continued to participate in Part B of the study which involved up to 12 weeks of use of the DEKA Arm at home, with in-person re-assessments every 4 weeks.

### Participant description

Participants were eligible for Part A (in-laboratory training), if they were at least 18 years old, had an upper limb amputation at the TR, TH, shoulder disarticulation or scapulothoracic level, and had sufficient control options available to them (e.g. myoelectric and /or active control over one or both feet for IMU use), to operate the functions of the DEKA Arm. Participants were excluded from Part A if their amputation level or skin condition prohibited prosthesis fitting or if they had serious health conditions that the study staff believed might limit their participation. At the completion of in-lab training, the study Principal Investigator (PI), in consultation with the study staff, determined which participants were appropriate for continuing to the home trial (Part B) using a priori criteria. Eligible subjects were required to demonstrate independent use of the prosthesis in laboratory and community settings. Subjects were also required to demonstrate at least fair functional performance with the DEKA Arm and consistent safety awareness and sound judgement when operating or troubleshooting minor technical issues with the prosthesis.

### Data collection

Tests and surveys administered at baseline, at the conclusion of Part A, and at the conclusion of Part B are described below.

#### Part A and Part B

Survey items administered at the end of Parts A and B asked participants to indicate if they wanted to receive a DEKA Arm in the future. Participants were asked to rate their skill level using the DEKA Arm, their perception of the weight of the DEKA Arm, and the comfort of the socket. If they were prosthesis users at baseline they were also asked if there were activities that they preferred doing with the DEKA Arm rather than with their own prosthesis, and whether there were activities that they could do with the DEKA Arm but could not do with their own prosthesis; as well as whether the reverse was true (activities preferred doing or could do with their own prosthesis but not the DEKA Arm). The Trinity Amputation and Prosthesis Experience Scales (TAPES) Satisfaction scale is a 10-item self-report measure. It was administered at baseline and conclusion of Parts A and B. Using a 5-point satisfaction rating (1 = Dissatisfied to 5 = Very Satisfied), participants rated overall satisfaction with characteristics such as reliability, comfort, fit, and cosmesis of a device.

#### Part A only

Survey items administered only at the end of Part A asked the subject if they felt that they had enough, too much or just the right amount of training. Additionally at the end of Part A, two measures developed for this study were administered. These were the 45-item DEKA Ease of Use Measure (Usability) and the 58-item DEKA Satisfaction Measure (Satisfaction) developed for this study and collected at the end of Part A. Items in the Usability scale were scored on a 6 point scale (1 = unable, 2 = very difficult, 3 = difficult, 4 = neither easy nor difficulty, 5 = easy, 6 = very easy). Items in the Satisfaction scale were scored using a 7-point scale (1 = very unhappy, 2 = unhappy, 3 = mostly dissatisfied, 4 = mixed, 5 = mostly satisfied, 6 = happy, 7 = very happy). Content of these item sets were based, in part, on the items that were used in the VA study to Optimize the DEKA Arm. [17]

#### Development and validation of usability and satisfaction

In order to determine subscale content and examine structural validity of the proposed subscales, we conducted an evaluation using preliminary data from 31 participants. First, items from the measures were grouped by general content category to determine if they could be aggregated to form subscales. We followed a commonly used method of examining item-test correlations and Cronbach’s alphas (a sample-dependent measure of internal consistency) to establish reliability of these subscales.[24]

Items with low item-test correlations (r<0.50) were removed from sub-scales. Items were also removed from any subscale with a Cronbach alpha <0.60 if their removal improved the Cronbach alpha. Preliminary analyses resulted in 8 Ease of Use subscales and 7 additional separate items that did not fit into subscales. The number of items within each subscale and Cronbach’s alphas of the 8 subscales were: Overall (15 items, α = 0.93), Batteries (4 items, α = 0.74), Cosmetic Covering (4 items, α = 0.65), IMUs (6 items, α = 0.86), SC Arm (2 items, α = 0.93), Suspension (3 items, α = 0.83), Tactor (3 items (α = 1.00) and Other Controls (2 items, α = 0.73).

Preliminary analyses resulted in 10 Satisfaction subscales and 5 items that did not fit into any subscales. The number of items within and Cronbach’s alphas of the 10 subscales were: Overall (21 items, α = 0.94), Batteries (4 items, α = 0.75), Cosmetic Covering (4 items, α = 0.76), EMGs (2 items, α = 0.96), IMUs (7 items, α = 0.88), Overall Cosmesis (3 items, α = 0.87), SC Arm (2 items, α = 0.92) Suspension (5 items, α = 0.96), Tactor (3 items (α = 1.01) and Other Controls (2 items, α = 0.68).

#### Part B only

Survey items administered at the end of Part B asked prosthesis users a set of questions about which they preferred: the DEKA Arm or their own prosthesis. These questions addressed: DEKA hand function, controls, weight, wrist function, look of the hand, look of the whole system, socket fit/comfort, using the DEKA Arm, and elbow / shoulder function (HC and SC users only). Subjects were also asked to indicate how necessary the DEKA Arm was for maintaining their quality of life, maintaining their independence, and improving their quality of life and independence.

### Data analysis

Descriptive statistics were used to characterize responses to survey items and measures for all subjects who completed Part A, and for those who completed both Parts A and Part B of the study. Results at the End of Part A and B were compared for those 18 subjects who completed both portions of the study. Preferences for various aspects of the DEKA Arm compared to current prosthesis were compared using Wilcoxon signed-rank, McNemar and paired t-tests for ordinal, dichotomous and continuous outcomes. [25, 26]

Responses to survey questions that asked prosthesis users to compare the DEKA arm to their own prosthesis were dichotomized into two categories: ‘disagree’ or ‘neither agree nor disagree’, and ‘agree’. Responses to questions about necessity of the prosthesis for improving or maintaining quality of life were dichotomized into two categories: not at all or slightly necessary, and moderately/quite a bit/extremely necessary. Two-sided Binomial tests were used to test the hypothesis that there would be no difference (i.e. that proportions for both responses are 50%) in preferences for the DEKA Arm and current prosthesis.

To evaluate concurrent validity of the satisfaction and usability scales we developed, we examined relationships between the subscales that we developed and the TAPES scale using Spearman correlations.

To determine if there were differences in usability and satisfaction by configuration level of the DEKA Arm, Kruskal Wallis analyses were performed to compare scores for all completers of Part A by configuration level. To determine whether there might be differences in responses for those subjects who were not likely to be adopters of the DEKA Arm (those who did not continue to the home use portion of the study) and those who did, responses to surveys and scores of measures were compared for the 18 participants who completed both Parts A and Part B and the 14 participants who did not complete Part B, using Wilcoxon rank-sum tests. To determine which aspects of satisfaction and usability were most strongly related to a desire to receive a DEKA Arm in the future we examined relationships between the satisfaction and usability subscales and the question ‘Do you want a DEKA Arm’ using Spearman correlations.

## Results

**Fig 1:**
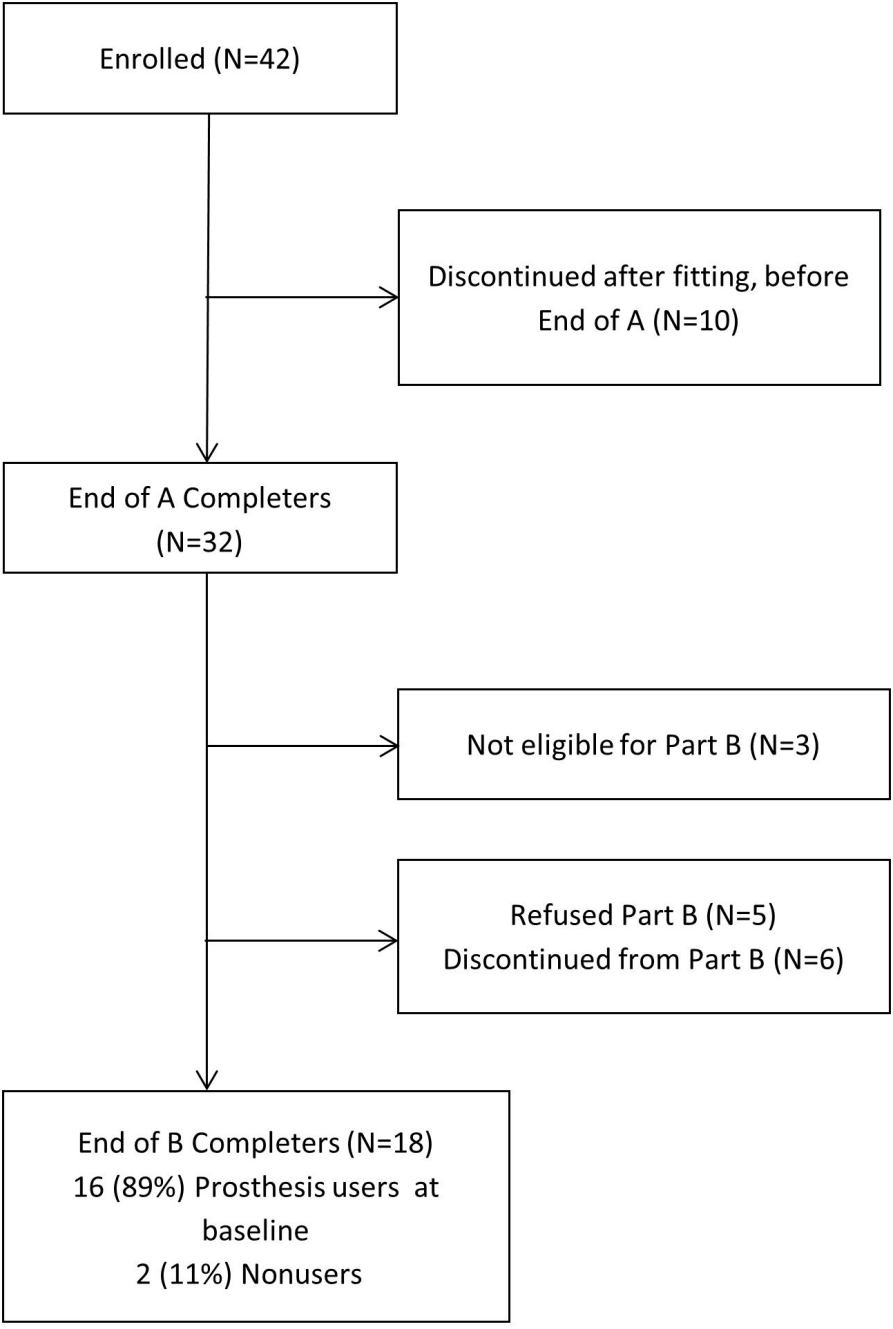
Study enrollment, attrition and completion.

Fig 1 shows the flow of participants through the study. Characteristics of the 32 participants (mn age = 45 ± 15; 94% male) who completed Part A and the 18 participants (mn age = 45 ± 15; 81% male) who completed both Parts A and B of the study are shown in Table 1. Fifty percent of the full sample had an amputation at the TR level, 38% had a TH amputation and 13% an amputation at the shoulder level. Fifty percent of participants used an RC, 25% used an HC and another 25% used an SC DEKA Arm. Fifty-six percent of participants who completed Part B had an amputation at the TR level, 39% at the TH level and 6% at the Shoulder level.

**Table 1:** Characteristics of sample.

	Part A Completers (N = 32)	Part A & B Completers (N = 18)
	Mn (sd)	Mn (sd)
Age (years)	45.1 (15.2)	44.8 (15.4)
	**N (%)**	**N (%)**
Gender		
Male	30 (93.8)	16 (80.9)
Female	2 (6.3)	2 (11.1)
Race		
White	25 (78.1)	16 (88.9)
Black	4 (12.5)	2 (11.1)
Other	3 (9.4)	0 (0.0)
Amputation level		
Transradial	16 (50.0)	10 (55.6)
Transhumeral	12 (37.5)	7 (38.9)
Shoulder disarticulation/forequarter	4 (12.5)	1 (5.6)
Prescribed DEKA Arm level		
RC	16 (50.0)	10 (55.6)
HC	8 (25.0)	6 (33.3)
SC	8 (25.0)	2 (11.1)
Prosthesis User (Yes)	24 (75.0)	16 (88.9)

Responses to survey items asked at the end of Part A and Part B are shown in Table 2. There were no significant differences in responses to survey items (pertaining to desire to receive a DEKA Arm, self-rated skill in use, perception of weight, ratings of socket comfort, or preferences for DEKA Arm over personal prosthesis) administered at the end of Part A and the end of Part B (Table 2). However, 4 fewer subjects (22%), 1 RC and 3 HC users, expressed a desire to receive a DEKA Arm at the end of Part B (Table 3).

**Table 2:** Survey items administered at End of A and End of B.

	End of A All (N = 32)	End of A+ (N = 18)	End of B (N = 18)	
	N (%)	N (%)	N (%)	P*
Do you want to receive a DEKA Arm in the future?				0.13
No	5 (15.6)	0 (0.0)	2 (11.1)	
Maybe	5 (15.6)	2 (11.1)	4 (22.2)	
Yes	22 (68.8)	16 (88.9)	12 (66.7)	
Self-rated skill level using DEKA Arm				0.22
Very Poor	0 (0.0)	0 (0.0)	0 (0.0)	
Poor	1 (3.1)	0 (0.0)	0 (0.0)	
Fair	4 (12.5)	2 (11.1)	0 (0.0)	
Good	17 (53.1)	10 (55.6)	9 (50.0)	
Excellent	10 (31.3)	6 (33.3)	9 (50.0)	
Perception of weight of DEKA Arm				0.73
Very light	2 (6.3)	0 (0.0)	0 (0.0)	
Light	8 (25.0)	2 (11.1)	2 (11.1)	
A little heavy	9 (28.1)	5 (27.8)	5 (27.8)	
Heavy	10 (31.3)	5 (27.8)	6 (33.3)	
Very Heavy	3 (9.4)	6 (33.3)	5 (27.8)	
Rating of socket comfort				1.00
Could not tolerate	1 (3.1)	0 (0.0)	0 (0.0)	
Uncomfortable	3 (9.4)	0 (0.0)	1 (5.6)	
Tolerable	9 (28.1)	6 (33.3)	5 (27.8)	
Comfortable	12 (37.5)	8 (44.4)	6 (33.3)	
Very comfortable	7 (21.9)	4 (22.2)	6 (33.3)	
**PROSTHESIS USERS ONLY**	**N = 24**	**N = 16**	**N = 16**	
Activities prefer doing with DEKA Arm than with current prosthesis?				0.13
No	2 (8.7)	0 (0.0)	14 (25.0)	
Maybe	3 (13.0)	3 (18.8)	2 (12.5)	
Yes	18 (78.3)	13 (81.3)	10 (62.5)	
Were there any activities that you can do with the DEKA Arm that you cannot do with your current prosthesis?				1.00
No	3 (13.0)	2 (12.5)	2 (12.5)	
Yes	20 (87.0)	14 (87.5)	14 (87.5)	
Were there any activities that you could not do with the DEKA Arm that you are able to do with your current prosthesis?				1.00
No	12 (52.2)	9 (56.3)	9 (56.3)	
Yes	11 (47.8)	7 (43.8)	7 (43.8)	
	**Mn (sd)**	**Mn (sd)**	**Mn (sd)**	**t-test**
TAPES	3.5 (0.8)	3.7 (0.6)	3.8 (0.9)	0.7354

+ End of A scores for those who also completed Part B

*Wilcoxon signed-rank and McNemar tests for categorical and dichotomous survey responses comparing N = 18 subjects who completed surveys at end of Part A and end of Part B

**Table 3:** Desire to receive a DEKA Arm at End of A and End of B* by configuration level (N = 18).

	Radial configuration (N = 10)	Humeral configuration (N = 6)	Shoulder configuration (N = 2)	Total (N = 18)
**At End of A: Do you want to receive a DEKA Arm in the future?**	**N (%)**	**N (%)**	**N (%)**	**N (%)**
No	0 (0.0)	0 (0.0)	0 (0.0)	0 (0.0)
Maybe	2 (20.0)	0 (0.0)	0 (0.0)	2 (11.1)
Yes	8 (80.0)	6 (100.0)	2 (100.0)	16 (88.9)
**At End of B: Do you want to receive a DEKA Arm in the future?**	**N (%)**	**N (%)**	**N (%)**	**N (%)**
No	1 (10.0)	1 (16.7)	0 (0.0)	2 (11.1)
Maybe	2 (20.0)	2 (33.3)	0 (0.0)	4 (22.2)
Yes	7 (70.0)	3 (50.0)	2 (100.0)	12 (66.7)

*Data shown is only for participants who completed Part A and Part B activities, and does not represent the views of those who dropped out of study activities.

Responses of 16 prosthesis users to survey items comparing the DEKA Arm to their own prosthesis at the end of Part B are shown in Table 4. Fourteen (88%) preferred the overall function of the DEKA Arm system to their own prosthesis (P = 0.004), 13 (81%) preferred the function of the DEKA hand (p = 0.021) and 14 (88%) preferred the function of the DEKA wrist (p = 0.004). In contrast, 14 (88%) and 13 (81%) preferred the weight and the look of their own prosthesis to the DEKA Arm (p = 0.004, and p = 0.021, respectively). There were no significant differences in the proportion who preferred the controls, look of the hand, fit of the socket, enjoyment of using, elbow, and shoulder of the DEKA Arm as compared to those aspects of their own prosthesis. Seven (44%) said the DEKA Arm was either not at all or only slightly necessary for maintaining their independence, while 9 (56%) found it moderately, quite a bit, or extremely necessary (p = 0.804).

**Table 4:** Survey items completed by prosthesis users only, administered at the end of Part B.

Please compare the DEKA Arm to your primary prosthesis, if you have one.*	Prosthesis users End of B (N = 16)	Binomial Test H_0_:P = 0.5
	N (%)	Exact p-value
I like the function of the DEKA hand better.		**0.0213**
Disagree / Neither agree nor disagree	3 (18.8)	
Agree	13 (81.3)	
I like the controls of the DEKA Arm system better.		0.8036
Disagree / Neither agree nor disagree	7 (43.8)	
Agree	9 (56.3)	
I like the weight of the DEKA Arm system better.		**0.0042**
Disagree / Neither agree nor disagree	14 (87.5)	
Agree	2 (12.5)	
I like the function of the DEKA wrist better.		**0.0042**
Disagree / Neither agree nor disagree	2 (12.5)	
Agree	14 (87.5)	
I like the look of the DEKA hand better.		1.0000
Disagree	8 (50.0)	
Agree	8 (50.0)	
10f. I like the look of the whole DEKA Arm system better.		**0.0213**
Disagree	13 (81.3)	
Agree	3 (18.8)	
I like the socket fit and general comfort of the DEKA Arm more.		0.6072
Disagree	9 (60.0)	
Agree	6 (40.0)	
I enjoyed using the DEKA Arm more.		0.4545
Disagree	6 (37.5)	
Agree	10 (62.5)	
For DEKA elbow users: I like the function of the elbow better.		0.2891
Disagree	2 (25.0)	
Agree	6 (75.0)	
For DEKA shoulder users: I like the function of the shoulder and whole arm better.		1.0000
Disagree	2 (50.0)	
Agree	2 (50.0)	
I like the overall function of the DEKA Arm system better.		**0.0042**
Disagree	2 (12.5)	
Agree	14 (87.5)	
How necessary is the DEKA Arm to you for maintaining your quality of life?		0.8036
Not at all/Slightly	7 (43.8)	
Moderately/Quite a bit/Extremely	9 (56.3)	
How necessary is the DEKA Arm to you for maintaining your independence?		0.8036
Not at all/Slightly	7 (43.8)	
Moderately/Quite a bit/Extremely	9 (56.3)	
Since starting the home study, please rate how much using the DEKA Arm has contributed to improving your quality of life?		0.2101
Not at all/Slightly	5 (31.3)	
Moderately/Quite a bit/Extremely	11 (60.8)	
Since starting the home study, please rate how much using the DEKA Arm has contributed to improving your independence?		0.8036
Not at all/Slightly	7 (43.8)	
Moderately/Quite a bit/Extremely	9 (56.3)	

*Original response categories were dichotomized. Combined categories are indicated by /.

Spearman correlations were estimated between the DEKA Usability subscales, DEKA Satisfaction subscales and the TAPES satisfaction scale, administered at the end of Part A (data not shown). The Overall DEKA Usability subscale, and the Batteries Usability subscale were strongly correlated (r≥0.5; p<0.05) with the TAPES measure of prosthetic satisfaction. While the Usability of the Cosmetic Covering, IMU, Suspension, and Tactor subscales were moderately correlated (r = 0.3–0.5; p<0.05). DEKA Satisfaction subscales including the Overall, IMUs, Overall Cosmesis, and Suspension were strongly correlated (r≥0.5; p<0.05) with the TAPES. The Batteries, EMGs and Tactor subscales were moderately correlated (r = 0.3–0.5; p<0.05).

Scores of Usability and Satisfaction subscales and items (completed at the end of Part A) are shown in Tables 5 and 6. At the end of Part A (all subjects) the DEKA Arm Usability (Table 5) Overall, Battery, Cosmetic Covering, IMU, and Tactor subscales had an average rating of 5.2–5.3. The Cosmetic Covering, Other Controls, SC Arm Subscale and Suspension subscale were rated on average 4.7, 4.5, 4.9 and 4.9, respectively (between “neither easy nor difficult” and “easy”). The 4 lowest average scorings among individual usability items were rated below “neither easy nor difficult to use”: the full arm system (mn 4.5), wires/cables (mn 4.5), shoulder function (mn 4.4), Endpoint control (mn 4.6). At the end of Part A the DEKA Arm Satisfaction (Table 6) Overall, Batteries, Cosmetic Covering, EMGs, IMUs, Overall Cosmesis, SC Arm, Suspension, and Tactor subscales were rated 5.2–5.9, indicating that users were “happy” with their function. The lowest average score for individual items were rated “mixed” and included: the full arm system, weight of the arm (mn 4.1), wires/cables, appearance of the shoulder (mn 4.1); harnessing system (mn 4.6). One item, dynamic straps was rated 3.9 on average (“mostly dissatisfied”).

**Table 5:** Ease of Use subscales and item scores for participants who completed only Part A and those who completed Part A and Part B.

Subscales Items		End of A (All) (N = 32)		End of A (those who completed B) (N = 18)		End of A (B non-completer) (N = 14)	Wilcoxon rank-sum
	N	Mn (sd)	N	Mn (sd)	N	Mn (sd)	P
**Overall Subscale**	32	5.2 (0.6)	18	5.3 (0.5)	14	4.9 (0.7)	0.1133
DEKA arm function	32	4.9 (0.8)	18	5.2 (0.7)	14	4.6 (0.9)	**0.0388**
Full arm system	30	4.5 (1.1)	16	4.8 (0.9)	14	4.1 (1.2)	0.1879
Pinch grip	32	5.3 (0.6)	18	5.4 (0.6)	14	5.2 (0.6)	0.3241
Chuck grip	32	5.2 (0.7)	18	5.2 (0.7)	14	5.2 (0.6)	0.8856
Tool grip	32	5.1 (0.8)	18	5.3 (0.6)	14	4.8 (0.9)	0.1076
Power grip	32	5.5 (0.5)	18	5.5 (0.5)	14	5.4 (0.5)	0.7345
Switching between grips	32	5.1 (0.8)	18	5.1 (0.6)	14	5.0 (1.0)	0.8777
Wrist movements	32	5.2 (0.8)	18	5.3 (0.6)	14	4.9 (0.9)	0.1947
Rotation of forearm	29	5.3 (0.6)	18	5.5 (0.6)	11	5.2 (0.4)	0.0524
Elbow movements	14	5.3 (0.8)	8	5.5 (0.5)	6	5.0 (1.1)	0.4522
Wires, cables	31	4.5 (1.1)	18	5.0 (0.8)	13	3.8 (1.2)	**0.0059**
Wrist display—grip indicator	32	5.3 (1.0)	18	5.4 (0.8)	14	5.2 (1.3)	0.8757
Wrist display—error indicator	32	5.3 (0.9)	18	5.4 (0.6)	14	5.1 (1.2)	0.6301
Wrist display—battery indicator	31	5.4 (0.9)	18	5.5 (0.6)	13	5.2 (1.1)	0.5787
Standby feature	31	5.5 (0.5)	18	5.6 (0.5)	13	5.5 (0.5)	0.4809
**Batteries Subscale**	32	5.3 (0.6)	18	5.4 (0.6)	14	5.2 (0.7)	0.3836
Battery charger	32	5.6 (0.6)	18	5.7 (0.5)	14	5.4 (0.8)	0.3133
External battery life	31	4.9 (1.1)	18	5.0 (1.0)	13	4.7 (1.2)	0.5081
Internal battery life	17	5.3 (0.8)	9	5.6 (0.7)	8	5.0 (0.8)	0.1742
Internal battery charging	17	5.5 (0.5)	9	5.7 (0.5)	8	5.4 (0.5)	0.3469
**Cosmetic Covering Subscale**	31	4.7 (0.7)	17	5.0 (0.6)	14	4.4 (0.8)	0.0569
Hand covering	26	4.8 (0.8)	13	4.9 (0.8)	13	4.6 (0.9)	0.4346
Material of hand cover	28	4.8 (1.1)	15	5.1 (0.7)	13	4.4 (1.4)	0.2163
Finger nails	30	4.7 (0.8)	17	4.9 (0.8)	13	4.4 (0.8)	0.0883
**IMUs Subscale**	32	5.3 (0.6)	18	5.3 (0.6)	14	5.2 (0.7)	0.3778
IMU controls	31	5.0 (0.9)	18	5.2 (0.6)	13	4.7 (1.2)	0.3211
Wrist display—walk detect	31	5.0 (1.0)	18	5.3 (0.7)	13	4.6 (1.3)	0.1274
Walk detect feature	32	5.4 (0.6)	18	5.4 (0.6)	14	5.4 (0.6)	0.7541
IMU battery charger	29	5.4 (0.7)	17	5.5 (0.6)	12	5.4 (0.8)	1.0000
IMU LED walk detect lights	30	5.4 (0.7)	18	5.3 (0.7)	12	5.4 (0.7)	0.7636
IMU LED battery level lights	31	5.4 (0.7)	18	5.3 (0.7)	13	5.4 (0.7)	0.9648
**SC Arm Subscale**	8	4.5 (1.2)	2	5.3 (0.4)	6	4.3 (1.3)	0.3571
Shoulder function	8	4.4 (1.3)	2	5.0 (0.0)	6	4.2 (1.5)	0.6429
Endpoint control	8	4.6 (1.2)	2	5.5 (0.7)	6	4.3 (1.2)	0.3571
**Suspension Subscale**	31	4.9 (0.8)	18	4.8 (0.9)	13	5.1 (0.8)	0.2603
Putting on socket and harness	31	4.7 (1.0)	18	4.7 (0.9)	13	4.8 (1.1)	0.6150
Taking off socket and harness	31	5.2 (1.0)	18	4.9 (1.1)	13	5.5 (0.9)	**0.0317**
Harnessing system	16	4.7 (1.0)	8	4.9 (1.1)	8	4.5 (0.9)	0.5114
**Tactor Subscale**	28	5.3 (0.8)	17	5.4 (0.9)	11	5.3 (0.6)	0.4754
Vibration sensors pressure	19	5.2 (1.1)	10	5.1 (1.3)	9	5.2 (0.8)	0.9541
Vibration sensors mode change	24	5.4 (0.8)	14	5.4 (0.9)	10	5.4 (0.7)	0.4626
Vibration sensors grip change	27	5.3 (0.8)	16	5.4 (0.9)	11	5.3 (0.6)	0.4976
**Other Controls Subscale**	22	4.9 (0.9)	12	5.2 (0.6)	10	4.6 (1.1)	0.1570
Other controls	12	4.8 (1.4)	7	5.4 (0.5)	5	4.0 (1.9)	0.2247
Inflatable bladders–	16	5.1 (0.6)	9	5.1 (0.6)	7	5.0 (0.6)	0.9567
**Individual items**							
Myoelectric controls	25	5.4 (0.9)	16	5.6 (0.5)	9	5.1 (1.3)	0.6987
VRE software	30	4.9 (1.0)	18	4.9 (1.1)	12	4.8 (1.0)	0.5675
Lateral pinch grip	32	5.3 (0.7)	18	5.4 (0.5)	14	5.2 (0.8)	0.5246
Rotation of upper arm	9	4.9 (0.9)	3	5.3 (0.6)	6	4.7 (1.1)	0.5119
Dynamic straps	7	4.9 (0.7)	1	4.0 (-)	6	5.0 (0.6)	0.4286
Dynamic socket controller	1	6.0 (-)	1	6.0 (-)	0	-	-
External Battery Life	31	5.4 (0.7)	17	5.4 (0.6)	14	5.3 (0.8)	0.8140

**Table 6:** Satisfaction subscales and item scores for participants who completed only Part A and those who completed Part A and Part B.

Subscales—Items		End of A (All) (N = 32)		End of A (B completer) (N = 18)		End of A (B non-completer) (N = 14)	Wilcoxon rank-sum
	N	Mn (sd)	N	Mn (sd)	N	Mn (sd)	P
**Overall**	32	5.4 (0.9)	18	5.8 (0.7)	14	5.0 (1.0)	**0.0165**
DEKA arm function	31	5.3 (1.4)	17	5.8 (1.0)	14	4.7 (1.6)	**0.0468**
Full arm system	32	4.5 (1.7)	18	4.9 (1.6)	14	4.1 (1.9)	0.1890
Hardware reliability	32	5.2 (1.4)	18	5.7 (0.9)	14	4.6 (1.7)	0.0604
Speed of hand open/close	31	6.0 (1.0)	18	6,2 (0.8)	13	5.7 (1.2)	0.1853
Pinch grip	32	6.0 (0.9)	18	6.2 (0.7)	14	5.7 (1.0)	0.2093
Chuck grip	32	5.7 (1.0)	18	6.8 (0.8)	14	5.3 (1.1)	0.0696
Lateral pinch grip	32	5.8 (1.2)	18	6.1 (0.9)	14	5.5 (1.5)	0.2470
Tool grip	32	5.3 (1.4)	18	5.7 (1.1)	14	4.9 (1.5)	0.0878
Power grip	32	5.9 (0.9)	18	6.2 (0.7)	14	5.6 (1.1)	0.1661
Switching between grips	32	5.2 (1.2)	18	5.6 (1.0)	14	5.0 (1.4)	0.2297
Wrist movements	32	5.5 (1.1)	18	5.7 (1.1)	14	5.2 (1.2)	0.1906
Rotation of forearm	29	5.6 (1.0)	16	5.8 (1.0)	13	5.4 (1.1)	0.2608
Elbow movements	14	5.6 (1.2)	8	5.6 (1.1)	6	5.5 (1.4)	1.0000
Rotation of upper arm	9	4.8 (1.8)	3	5.7 (0.6)	6	4.3 (2.1)	0.4405
Weight of arm	32	4.1 (1.7)	18	4.6 (1.4)	14	3.5 (1.8)	0.1097
Wires, cables	32	4.3 (1.8)	18	4.9 (1.4)	14	3.5 (2.0)	0.0639
Wrist display—grip indicator	32	5.7 (1.2)	18	6.0 (1.0)	14	5.3 (1.4)	0.1728
Wrist display—error indicator	32	5.7 (1.2)	18	6.0 (0.8)	14	5.2 (1.5)	0.1656
Wrist display—battery indicator	31	5.7 (1.2)	18	5.9 (0.8)	13	5.5 (1.5)	0.6983
External Battery Life	32	5.7 (1.1)	18	5.8 (0.8)	14	5.5 (1.5)	0.9928
Standby feature	31	6.0 (0.7)	18	6.2 (0.7)	13	5.8 (0.7)	0.2118
**Batteries**	32	5.6 (1.1)	18	5.8 (0.8)	14	5.2 (1.4)	0.1741
Battery charger	32	5.7 (1.4)	18	5.9 (0.9)	14	5.4 (1.9)	0.6029
External battery life	30	5.1 (1.3)	17	5.5 (1.0)	13	4.5 (1.5)	**0.0355**
Internal battery life	18	5.9 (0.9)	10	6.0 (1.1)	8	5.9 (0.8)	0.7183
Internal battery charging	16	6.2 (0.8)	8	6.4 (0.7)	8	6.0 (0.8)	0.4443
**Cosmetic Covering**	32	5.3 (1.2)	18	5.6 (0.7)	14	4.9 (1.5)	0.2924
Hand covering	31	5.5 (1.1)	18	5.7 (1.1)	13	5.2 (1.1)	0.1621
Hand cover durability	31	5.4 (1.3)	18	5.7 (1.0)	13	5.0 (1.5)	0.2604
Material of hand cover	32	5.3 (1.3)	18	5.4 (1.0)	14	5.1 (1.7)	0.8778
Finger nails	30	5.3 (1.3)	18	5.6 (1.1)	12	4.8 (1.5)	0.1380
**EMGs**	25	5.7 (1.4)	16	5.9 (0.9)	9	5.3 (2.1)	0.9679
Myoelectric controls	25	5.6 (1.4)	16	5.8 (1.0)	9	5.4 (1.9)	0.8747
EMG speed	25	5.7 (1.5)	16	6.1 (0.9)	9	5.2 (2.3)	0.8186
**IMUs**	32	5.7 (0.8)	18	5.8 (0.8)	14	5.5 (0.8)	0.2948
IMU controls	30	5.2 (1.5)	18	5.3 (1.5)	12	5.2 (1.6)	0.8716
IMU speed	30	5.7 (1.1)	17	6.0 (0.9)	13	5.3 (1.2)	0.0679
Wrist display—walk detect	31	5.2 (1.3)	18	5.6 (1.0)	13	4.5 (1.3)	0.0239
Walk detect feature	32	5.8 (0.9)	18	5.9 (0.9)	14	5.6 (1.0)	0.4078
IMU battery charger	31	6.1 (0.7)	18	6.1 (0.8)	13	6.1 (0.5)	0.8140
IMU LED walk detect lights	31	5.8 (0.9)	18	5.8 (0.9)	13	5.7 (0.9)	0.8177
IMU LED battery level lights	31	5.8 (0.9)	18	5.9 (1.0)	13	5.8 (0.9)	0.7061
**Overall Cosmesis**	32	5.4 (1.3)	18	5.4 (1.4)	14	5.3 (1.3)	0.6575
DEKA arm appearance	31	4.7 (1.8)	17	4.9 (1.6)	14	4.5 (2.0)	0.5924
DEKA hand shape	32	5.8 (1.2)	18	5.7 (1.3)	14	5.8 (1.1)	0.9672
DEKA hand size	32	5.6 (1.5)	18	5.6 (1.6)	14	5.6 (1.4)	0.8168
**SC Arm**	8	4.9 (1.8)	2	5.5 (0.7)	6	4.7 (2.0)	0.8571
Shoulder function	8	4.8 (2.3)	2	5.5 (0.7)	6	4.5 (2.6)	1.0000
Endpoint control	8	5.0 (1.5)	2	5.5 (0.7)	6	4.8 (1.7)	0.7500
**Suspension**	32	5.2 (1.3)	18	5.7 (1.0)	14	4.9 (1.5)	0.1824
Putting on socket and harness	31	5.1 (1.4)	18	5.3 (1.3)	13	4.8 (1.4)	0.2897
Comfort of socket	31	5.6 (1.5)	17	5.9 (1.0)	14	5.4 (1.9)	0.7139
Harnessing system	18	4.6 (1.6)	8	4.9 (1.1(	10	4.3 (1.9)	0.5925
Dynamic straps	7	3.9 (2.0)	1	4.0 (-)	6	3.8 (2.2)	1.0000
Stability of socket	31	5.7 (1.4)	18	6.1 (1.0)	13	5.3 (1.8)	0.3424
**Tactor**	29	5.5 (1.5)	17	5.5 (1.3)	12	5.5 (1.7)	0.9737
Vibration sensors pressure	20	5.3 (1.9)	10	5.4 (1.8)	10	5.1 (2.1)	0.9127
Vibration sensors mode change	24	5.5 (1.5)	14	5.4 (1.3)	10	5.5 (1.7)	0.7060
Vibration sensors grip change	27	5.5 (1.4)	16	5.6 (1.3)	11	5.5 (1.6)	0.9291
**Other controls**	22	4.9 (0.9)	12	5.2 (0.6)	10	4.6 (1.1)	0.1570
Other controls	12	4.8 (1.4)	7	5.4 (0.5)	5	4.0 (1.9)	0.2247
Inflatable bladders	16	5.1 (0.6)	9	5.1 (0.6)	7	5.0 (0.6)	0.9567
**Individual items**							
VRE software	30	4.7 (1.4)	18	4.7 (1.4)	12	4.7 (1.4)	0.9404
Taking off socket and harness	31	5.7 (1.2)	18	5.7 (1.3)	13	5.8 (1.0)	0.4481
Appearance of shoulder	7	4.1 (1.6)	2	3.0 (0.0)	5	4.6 (1.7)	0.1905
Dynamic socket controller	1	7.0 (-)	1	7.0 (-)	0	-	
Level of waterproofing	32	5.0 (1.3)	18	4.3 (1.5)	14	5.1 (0.9)	0.8478

There were no statistically significant differences in scores of the DEKA Usability subscales for participants who completed Parts A and B and those who completed only Part A (Table 5). However 2 individual Usability items were rated higher by those participants who completed Part B as compared to participants who did not: DEKA Arm function mn 5.2 for completers, mn 4.6 for non-completers(p = 0.039), and wires/ cables mn 5.0 for completers, mn 3.8 for non-completers (0.006). One item, taking off socket and harness was rated significantly (p = 0.032) higher by those who did not complete Part B (mn 4.9) as compared to those who completed both Parts A and B (mn 5.5). There was only one subscale from the DEKA Satisfaction subscales with a statistically significant difference in ratings by study completion status (Table 6). The Overall subscale was rated significantly (p = 0.0165) higher by completers of Part B (mn 5.8) than non-completers (mn 5.0). Additionally, there were significant differences in the satisfaction rating for two individual items. Participants who completed Part B rated the DEKA function and the external battery life (mn 5.8, 5.8, 5.5) higher than those who did not complete Part B (mn 5.0; 4.7; 4.5, respectively).

Results of Kruskal-Wallis comparisons of DEKA Usability ratings for all completers of Part A by configuration level (Table 7), found significant differences in the usability ratings for 3 items: tactor; vibration sensors pressure; and vibration sensors grip change. Participants using an HC Arm rated usability of the tactor, vibration sensors pressure and vibration sensors for grip, higher (mn 5.8; 5.7; 5.9, respectively) as compared to subjects using an RC (mn 5.4; 5.4; 5.3, respectively) and Shoulder (mn 4.9; 4.4; 4.9, respectively). However, there were no significant differences for the satisfaction subscales by configuration level (results not shown).

**Table 7:** Usability ratings by level of DEKA Arm configuration level.

Scales	Radial configuration(N = 16)	Humeral configuration (N = 8)	Shoulder configuration (N = 8)	
	Mn (sd)	Mn (sd)	Mn (sd)	KW P
**Overall**	5.2 (0.6)	5.2 (0.7)	5.0 (0.6)	0.6537
DEKA arm function	6.1 (0.7)	4.6 (1.1)	4.9 (0.8)	0.5508
Full arm system	4.5 (1.2)	4.4 (1.1)	4.4 (1.1)	0.9853
Pinch grip	5.4 (0.6)	5.4 (0.7)	5.3 (0.5)	0.7855
Chuck grip	5.2 (0.7)	5.3 (0.9)	5.3 (0.5)	0.9375
Tool grip	4.9 (0.8)	5.3 (0.9)	5.1 (0.6)	0.6209
Power grip	5.4 (0.5)	5.6 (0.5)	5.4 (0.5)	0.5786
Switching between grips	5.1 (0.8)	5.0 (0.5)	5.1 (1.1)	0.7133
Wrist movements	4.9 (0.9)	5.5 (0.5)	5.3 (0.5)	0.2753
Rotation of forearm	5.3 (0.5)	5.5 (0.8)	5.3 (0.5)	0.4796
Elbow movements	-	5.5 (0.5)	5.0 (1.1)	0.3865
Wires, cables	4.5 (1.3)	4.7 (1.1)	4.4 (0.7)	0.7358
Wrist display—grip indicator	5.6 (0.9)	5.1 (1.1)	5.1 (1.1)	0.3784
Wrist display—error indicator	5.6 (0.8)	5.1 (1.0)	4.9 (1.0)	0.1005
Wrist display—battery indicator	5.6 (0.8)	5.1 (1.0)	5.0 (0.8)	0.0789
Standby feature	5.7 (0.5)	5.5 (0.5)	5.3 (0.5)	0.0880
**Batteries**	5.3 (0.7)	5.4 (0.6)	5.1 (0.6)	0.7001
Battery charger	5.6 (0.7)	5.8 (0.5)	5.5 (0.5)	0.6290
External battery life	5.0 (1.1)	4.9 (1.0)	4.6 (1.2)	0.7187
Internal battery life	6.0 (0.0)	5.4 (0.7)	5.0 (0.8)	0.2263
Internal battery charging	6.0 (0.0)	5,5 (0.5)	5.4 (0.5)	0.3738
**Cosmetic Covering**	4.8 (0.8)	4.8 (0.7)	4.4 (0.8)	0.3496
Hand covering	4.8 (0.6)	5.0 (1.0)	4.5 (1.0)	0.6582
Material of hand cover	4.9 (1.1)	5.0 (0.6)	4.2 (1.3)	0.3207
Finger nails	4.9 (0.9)	4.7 (0.8)	4.3 (0.8)	0.4002
**IMUs**	5.4 (0.6)	5.3 (0.6)	4.9 (0.6)	0.1728
IMU controls	4.9 (1.0)	4.9 (1.0)	5.1 (0.6)	0.9247
Wrist display—walk detect	5.4 (0.9)	4.9 (0.8)	4.5 (1.2)	0.0838
Walk detect feature	5.5 (0.6)	5.5 (0.5)	5.1 (0.6)	0.3279
IMU battery charger	5.6 (0.6)	5.5 (0.5)	5.0 (0.9)	0.2527
IMU LED walk detect lights	5.5 (0.6)	5.5 (0.5)	5.0 (0.8)	0.3285
IMU LED battery level lights	5.5 (0.6)	5.5 (0.5)	5.0 (0.8)	0.2565
**SC Arm**	-	-	4.5 (1.2)	-
Shoulder function	-	-	4.4 (1.3)	-
Endpoint control	-	-	4.6 (1.2)	-
**Suspension**	5.0 (0.7)	5.1 (0.8)	4.6 (1.1)	0.6543
Putting on socket and harness	5.0 (0.5)	4.7 (1.1)	4.3 (1.4)	0.4655
Taking off socket and harness	5.2 (1.1)	5.4 (0.5)	4.9 (1.2)	0.7541
Harnessing system	4.0 (0.0)	5.0 (1.3)	4.6 (0.9)	0.3993
**Tactor**	5.4 (0.9)	5.8 (0.4)	4.9 (0.6)	**0.0227**
Vibration sensors pressure	5.4 (1.2)	5.7 (0.6)	4.4 (0.5)	**0.0335**
Vibration sensors mode change	5.3 (1.0)	5.9 (0.4)	5.0 (0.8)	0.0673
Vibration sensors grip change	5.3 (0.9)	5.9 (0.4)	4.9 (0.6)	**0.0196**
Other Controls	4.5 (1.1)	5.4 (0.5)	5.1 (0.4)	0.0905
Other controls	4.0 (1.9)	5.7 (0.6)	5.3 (0.5)	0.2601
Inflatable bladders–	4.9 (0.6)	5.3 (0.5)	5.3 (0.5)	0.4386
**Individual items**				
Myoelectric controls	5.6 (0.5)	5.6 (0.5)	4.6 (1.5)	0.3775
VRE software	5.0 (1.0)	5.0 (1.0)	4.5 (1.1)	0.4462
Lateral pinch grip	5.3 (0.8)	5.5 (0.5)	5.3 (0.5)	0.6312
Rotation of upper arm	-	6.0 (-)	4.8 (0.9)	0.1356
Dynamic straps	4.5 (0.7)	4.0 (-)	5.3 (0.5)	0.1695
Dynamic shoulder controller	-	6.0 (-)	-	-
External Battery Life	5.5 (0.6)	5.3 (0.7)	5.1 (0.9)	0.5365

Spearman correlation analyses found that user’s desire to receive a DEKA Arm in the future was strongly and significantly associated with the TAPES satisfaction scale (r = 0.75), our Overall Satisfaction scale (r = 0.56), and our Satisfaction with Suspension scale (r = 0.51), and moderately associated with the Overall Usability scale (r = 0.39), Satisfaction with Cosmesis (r = 0.38), and Satisfaction with the IMU subscale (r = 0.36). No other subscales or individual items were significantly associated with desire to receive a DEKA Arm in the future.

## Discussion

Little research has been conducted to understand if and how attitudes towards upper limb prosthetic technology change once users have the opportunity to use a device outside of a supervised clinical setting and attempt to integrate it into their daily lives at home and in the community. Our study described user perceptions of the Gen 3 DEKA Arm at the end of in-laboratory training and after a trial of home use and compared some user perceptions over time. Our findings augment those of prior research on user perceptions of the Gen 3 DEKA Arm in an in-laboratory study only. [17, 27] We found that the proportion of participants who completed in-laboratory training who wanted to receive a DEKA Arm or might want to receive one (88.9% and 11% respectively) after completing in-laboratory training mirrored the proportion reported in earlier work (64% and 18% respectively). Nevertheless, we recognize that these findings need to be interpreted cautiously, given that 21% of 42 study participants withdrew prior to completing in-laboratory training and many reasons for attrition were related to user perceptions about the DEKA Arm. [18, 19]Thus, the true proportion of upper limb amputees who might desire a DEKA Arm is likely to be lower than what we found in this and earlier studies. One third of participants queried at the end of Part B thought that the Gen 3 DEKA Arm that they used was not yet ready for commercialization, and still needed refinements.

The findings reported here corroborate earlier observations that the sub-group of subjects who participated in the home trial of the device (Part B) were more enthusiastic about wanting a DEKA Arm at the end of in-laboratory training (Part A) (89% indicated yes, and 11% maybe) as compared to the overall group of participants who completed Part A training. [27] Not surprisingly, overall satisfaction with the DEKA Arm (as measured by the TAPES) at the end of in-laboratory training was strongly associated with an expressed desire to receive a DEKA Arm at the end of Part B. Additionally, those who were retained in the study and completed all home activities (and thus might be considered more likely adopters of the DEKA Arm in the future), had higher ratings of the DEKA Arm function item in the Usability and Satisfaction subscales, as well as significantly higher ratings of the Satisfaction with the Overall subscale item, Satisfaction with the external battery life item, and the wires/cables usability item as compared to those who withdrew without completing. Further, a general trend was observed with lower scores on most individual items for those participants who did not complete Part B as compared to those who did. In prior work involving a smaller sample of Gen 3 DEKA Arm Users, it was concluded that the Gen 3 DEKA Arm would benefit from additional optimization to decrease its weight, minimize external cables and wires, and improve the hand covering, and fingernails. [17] The current study confirms that participants who did not complete Part B in the current study also unfavorably viewed these aspects of the DEKA Arm usability (wires and cables was the single lowest rated usability item in that group) and satisfaction (weight, wires and cables were the lowest rated satisfaction items). Our findings also confirm the overall positive regard for IMU usability and satisfaction that we reported in our earlier work evaluating user perspectives on foot controls. [21] Overall, the participants in the Home Study rated that IMUs as “easy to use” and were happy with them as a control method.

While our earlier analyses found that persons who were prosthesis users and did not have a history of musculoskeletal problems were more likely to reach study completion, the findings reported here suggest that users’ attitudes at the end of in-laboratory training are strong indicators of participants’ ultimate willingness to adopt an advanced device like the DEKA Arm. These findings illustrate the utility of having an in-laboratory trial period of the DEKA Arm before a patient and provider make a decision about appropriateness of a prescription and purchase.

That said, some of our study participants changed their minds about their desire to receive a DEKA Arm after bringing it home. Although the Wilcoxon signed-ranks comparison between End of A and End of B was not statistically significant (p = 0.13), there were 4 fewer participants (1 RC and 3 HC users) who expressed a desire to receive a DEKA Arm after the home use experience, suggesting that the home use experience may have tempered the views of these participants. The 2 SC users who completed Part B indicated a consistent desire to receive a DEKA Arm, supporting previous findings that users with more proximal level amputation were more enthusiastic about the potential of the DEKA Arm. [27] Although we could not compare ratings on the usability and satisfaction subscales that we developed across time periods (because these were only administered at the end of Part A), we did not find statistically significant differences in overall satisfaction ratings of the TAPES satisfaction scale before and after the experience of home use. Further research exploring the qualitative data from this study may help to explain the reasons that some participants changed their views about the desirability of the DEKA Arm.

As reported, we found that usability ratings of two items were lower for those subjects who completed Part A but did not complete all home use activities: overall DEKA Arm function and wires/cables. In contrast ratings of ease donning and doffing were higher for the group of non-completers. This finding may suggest that ease of donning and doffing the socket itself is not weighed as heavily as the securing of the external battery or other accessories that requires wires and cables.

At the end of the home use period we found that those participants who had a personal prosthesis expressed mixed preferences about which device they preferred. Generally, these participants preferred the overall function of the DEKA Arm system, the DEKA hand, and DEKA wrist over their personal prostheses. However they preferred the appearance and weight of their own prosthesis to the DEKA Arm.

We found few differences in usability and satisfaction ratings by DEKA Arm configuration level. There were some differences in usability ratings of the tactor, with SC users rating it less usable and HC users rating the tactor more favorably as compared to RC users. This may be due to the location of the tactor on the torso of SC users, which arguably is less sensitive to vibratory stimuli as compared to the residual limb of the HC or RC users. Alternatively, lower ratings of the tactor, were associated with the greater cognitive burden required for SC users to operate the device. [23] Given this, these users may be less able to attend to vibratory inputs of the tactor. Further research is needed with larger samples and employing other methods (such as qualitative methods) to more fully understand the differences in user perception by configuration level.

This study had several limitations. First, the usability and satisfaction scales used were specific to this study. The content of these scales were based, in large part, on refinement of the content of scales used in an earlier study of the DEKA Arm.[17] We grouped items into scales based upon their content and the results of analyses of scale internal consistency and item fit. We believe that the scale items have face validity and that the subscales identified have concurrent validity, given their observed moderate to strong correlations with the TAPES satisfaction measure for most subscales. We established reliability through examining internal consistency, but recognize that measurement of Cronbach’s alpha can be considered a lower-bound estimate of reliability. [28] We were unable to examine test-retest reliability because we only administered the usability and satisfaction scales at one time point (end of Part A). This also made it impossible to track changes in participants’ ratings over time. Further studies are needed to examine test-retest reliability, validity, and responsiveness of these scales.

Another limitation is that our results may not be generalizable to all persons who might try the DEKA Arm; rather they are limited to the sample of persons who had completed a training protocol and all requirements of in-laboratory and home trials with the device. While we believe that some findings may be transferable to a wider population of persons with upper limb amputees, our findings may overestimate amputee’s perception of usability and satisfaction of the DEKA Arm, given that our earlier work found that those who did not complete all study activities had more negative views of the DEKA Arm and its features. [18]

## Conclusions

Thirty-two participants who completed in-laboratory training were asked if they wanted to receive a DEKA Arm in the future, and rated the usability of and satisfaction with various aspects of the DEKA Arm. The majority of study participants who completed Part A stated that they wanted to receive a DEKA Arm or might want to receive one in the future. At the end of the home use portion of the study, 88% of 16 participants who completed the study and were users of a prosthesis at baseline indicated that they preferred the overall function of the DEKA Arm and its wrist function and 81% preferred the DEKA hand to their own device. In contrast, 88% preferred the weight and 81% preferred the look of their own devices compared to the DEKA Arm. Participants rated most DEKA Arm features as “easy to use” and indicated that they were “happy” with them. The aspects of the DEKA Arm usability that were rated least favorably (“neither easy nor difficult to use”) included the full arm system, wires/cables, shoulder function, and Endpoint control. The items from the DEKA Satisfaction scales that users rated least favorably included: the full arm system, weight of the arm, wires, cables, appearance of the shoulder and harnessing system (all neither satisfied nor dissatisfied) and the dynamic straps (dissatisfied).

Satisfaction with various aspects of the DEKA Arm did vary by configuration level. However, participants using an HC Arm rated usability of the tactor, vibration sensors pressure and vibration sensors for grip, higher than those using an RC or shoulder.

Differences in satisfaction and usability ratings were observed between study completers and non-completers with the perceived overall usability of the DEKA Arm, usability of wires/cables, satisfaction with the DEKA Arm function, satisfaction with full arm system, and satisfaction with the external battery rated less favorably by study non-completers as compared to those who completed all home study activities. This suggests that a self-contained system which did not require external accessories, such as an external battery and associated cables, might have led to increased retention in the study. These findings suggest that an in-laboratory trial period of the device may be useful to allow patients to experience the DEKA Arm over a prolonged period of time prior to finalizing a recommendation for prescription. This type of trial period may, in the long-run, prevent abandonment of an expensive device.

## Supporting information

S1 Appendix Survey questions: DEKA Arm usability and satisfaction scales. (DOCX)
