# Supplementary material for: Perceptions of satisfaction, usability and desirability of the DEKA Arm before and after a trial of home use
Source: PLoS One. 2017 Jun 2;12(6):e0178640. doi: 10.1371/journal.pone.0178640 (PMC5456350; doi:10.1371/journal.pone.0178640)
Supplement: S1 Appendix — (DOCX) [file pone.0178640.s001.docx]

**Appendix A**

**INSTRUMENTS**

**1. DEKA ARM USABILITY SCALE**

**Administrator instructions**

For the following items, please ask the subject to rank how easy or difficult it was for them to use specific features of the DEKA Arm. Mark the box that best describes the subject’s opinion.

**Scoring**

Higher scores from 1-6, indicate greater ease of use.

Please enter NA=“Not applicable”, if the subject was not set up for a particular item. Please enter “Don’t Know”, as needed.

|  | **Unable**  **to do**  **1** | **Very difficult**  **2** | **Difficult**  **3** | **Neither difficult**  **nor easy**  **4** | **Easy**  **5** | **Very easy**  **6** | **Don’t**  **know** | **NA** |
| --- | --- | --- | --- | --- | --- | --- | --- | --- |
| **Overall** |  |  |  |  |  |  |  |  |
| DEKA arm function |  |  |  |  |  |  |  |  |
| Full arm system |  |  |  |  |  |  |  |  |
| Pinch grip |  |  |  |  |  |  |  |  |
| Chuck grip |  |  |  |  |  |  |  |  |
| Tool grip |  |  |  |  |  |  |  |  |
| Power grip |  |  |  |  |  |  |  |  |
| Switching between grips |  |  |  |  |  |  |  |  |
| Wrist movements |  |  |  |  |  |  |  |  |
| Rotation of forearm |  |  |  |  |  |  |  |  |
| Elbow movements |  |  |  |  |  |  |  |  |
| Wires, cables |  |  |  |  |  |  |  |  |
| Wrist display - grip indicator |  |  |  |  |  |  |  |  |
| Wrist display - error indicator |  |  |  |  |  |  |  |  |
| Wrist display - battery  indicator |  |  |  |  |  |  |  |  |
| Standby feature |  |  |  |  |  |  |  |  |
| **Batteries** |  |  |  |  |  |  |  |  |
| Battery charger |  |  |  |  |  |  |  |  |
| External battery life |  |  |  |  |  |  |  |  |
| Internal battery life |  |  |  |  |  |  |  |  |
| Internal battery charging |  |  |  |  |  |  |  |  |
| **Cosmetic covering** |  |  |  |  |  |  |  |  |
| Hand covering |  |  |  |  |  |  |  |  |
| Material of hand cover |  |  |  |  |  |  |  |  |
| Finger nails |  |  |  |  |  |  |  |  |
| **IMUs** |  |  |  |  |  |  |  |  |
| IMU controls |  |  |  |  |  |  |  |  |
| Wrist display - walk detect |  |  |  |  |  |  |  |  |
| Walk detect feature |  |  |  |  |  |  |  |  |
| IMU battery charger |  |  |  |  |  |  |  |  |
| IMU LED walk detect lights |  |  |  |  |  |  |  |  |
| IMU LED battery level lights |  |  |  |  |  |  |  |  |
|  |  |  |  |  |  |  |  |  |
|  | **Unable**  **to do**  **1** | **Very difficult**  **2** | **Difficult**  **3** | **Neither difficult**  **nor easy**  **4** | **Easy**  **5** | **Very easy**  **6** | **Don’t**  **know** | **NA** |
| **SC Arm** |  |  |  |  |  |  |  |  |
| Shoulder function |  |  |  |  |  |  |  |  |
| Endpoint control |  |  |  |  |  |  |  |  |
| **Suspension** |  |  |  |  |  |  |  |  |
| Putting on socket and harness |  |  |  |  |  |  |  |  |
| Taking off socket and harness |  |  |  |  |  |  |  |  |
| Harnessing system |  |  |  |  |  |  |  |  |
| **Tactor** |  |  |  |  |  |  |  |  |
| Vibration sensors pressure |  |  |  |  |  |  |  |  |
| Vibration sensors mode  change |  |  |  |  |  |  |  |  |
| Vibration sensors grip change |  |  |  |  |  |  |  |  |
| **Other controls** |  |  |  |  |  |  |  |  |
| Other controls |  |  |  |  |  |  |  |  |
| Inflatable bladders |  |  |  |  |  |  |  |  |
| **Misc Individual items** |  |  |  |  |  |  |  |  |
| Myoelectric controls |  |  |  |  |  |  |  |  |
| VRE software |  |  |  |  |  |  |  |  |
| Lateral pinch grip |  |  |  |  |  |  |  |  |
| Rotation of upper arm |  |  |  |  |  |  |  |  |
| Dynamic straps |  |  |  |  |  |  |  |  |
| Dynamic socket controller |  |  |  |  |  |  |  |  |
| External Battery Life |  |  |  |  |  |  |  |  |

**S1 Table. DEKA Arm Usability scale**

**2. DEKA ARM SATISFACTION SCALE**

**Administrator instructions**

For the following questions, please ask the subject to rate how happy or unhappy he/she was with each of the following features of DEKA Arm?

**Scoring**

Higher scores from 1-7, indicate greater Satisfaction.

Please enter NA=“Not applicable”, if the subject was not set up for a particular item.

|  | **Very**  **Unhappy**  **1** | **Unhappy**  **2** | | **Mostly dissatisfied**  **3** | **Mixed**  **4** | | | **Mostly satisfied**  **5** | | **Happy**  **6** | **Very Happy**  **7** | **NA** |
| --- | --- | --- | --- | --- | --- | --- | --- | --- | --- | --- | --- | --- |
| **Overall** |  |  | |  |  | | |  | |  |  |  |
| DEKA arm function |  |  | |  |  | | |  | |  |  |  |
| Full arm system |  |  | |  |  | | |  | |  |  |  |
| Hardware reliability |  |  | |  |  | | |  | |  |  |  |
| Speed of hand open/close |  |  | |  |  | | |  | |  |  |  |
| Pinch grip |  |  | |  |  | | |  | |  |  |  |
| Chuck grip |  |  | |  |  | | |  | |  |  |  |
| Lateral pinch grip |  |  | |  |  | | |  | |  |  |  |
| Tool grip |  |  | |  |  | | |  | |  |  |  |
| Power grip |  |  | |  |  | | |  | |  |  |  |
| Switching grips |  |  | |  |  | | |  | |  |  |  |
| Wrist movements |  |  | |  |  | | |  | |  |  |  |
| Rotation of forearm |  |  | |  |  | | |  | |  |  |  |
| Elbow movements |  |  | |  |  | | |  | |  |  |  |
| Rotation of upper arm |  |  | |  |  | | |  | |  |  |  |
| Weight of arm |  |  | |  |  | | |  | |  |  |  |
| Wires, cables |  |  | |  |  | | |  | |  |  |  |
| Wrist display - grip indicator |  |  | |  |  | | |  | |  |  |  |
| Wrist display - error indicator |  |  | |  |  | | |  | |  |  |  |
| Wrist display - battery  indicator |  |  | |  |  | | |  | |  |  |  |
| External Battery Life |  |  | |  |  | | |  | |  |  |  |
| Standby feature |  |  | |  |  | | |  | |  |  |  |
| **Batteries** |  |  | |  |  | | |  | |  |  |  |
| Battery charger |  |  | |  |  | | |  | |  |  |  |
| External battery life |  |  | |  |  | | |  | |  |  |  |
| Internal battery life |  |  | |  |  | | |  | |  |  |  |
| Internal battery charging |  |  | |  |  | | |  | |  |  |  |
| **Cosmetic Covering** |  |  | |  |  | | |  | |  |  |  |
| Hand covering |  |  | |  |  | | |  | |  |  |  |
| Hand cover durability |  |  | |  |  | | |  | |  |  |  |
| Material of hand cover |  |  | |  |  | | |  | |  |  |  |
| Finger nails |  |  | |  | |  |  | |  | |  |  |
| **EMGs** |  |  |  | |  | | |  | |  |  |  |
| Myoelectric controls |  |  |  | |  | | |  | |  |  |  |
| EMG speed |  |  |  | |  | | |  | |  |  |  |
| **IMUs** |  |  |  | |  | | |  | |  |  |  |
| IMU controls |  |  |  | |  | | |  | |  |  |  |
|  | **Very**  **Unhappy**  **1** | **Unhappy**  **2** | **Mostly dissatisfied**  **3** | | **Mixed**  **4** | | | **Mostly satisfied**  **5** | | **Happy**  **6** | **Very Happy**  **7** | **NA** |
| IMU speed |  |  |  | |  | | |  | |  |  |  |
| Wrist display - walk detect |  |  |  | |  | | |  | |  |  |  |
| Walk detect feature |  |  |  | |  | | |  | |  |  |  |
| IMU battery charger |  |  |  | |  | | |  | |  |  |  |
| IMU LED walk detect lights |  |  |  | |  | | |  | |  |  |  |
| IMU LED battery level lights |  |  |  | |  | | |  | |  |  |  |
| **Overall/Cosmesis** |  |  |  | |  | | |  | |  |  |  |
| DEKA arm appearance |  |  |  | |  | | |  | |  |  |  |
| DEKA hand shape |  |  |  | |  | | |  | |  |  |  |
| DEKA hand size |  |  |  | |  | | |  | |  |  |  |
| **SC Arm** |  |  |  | |  | | |  | |  |  |  |
| Shoulder function |  |  |  | |  | | |  | |  |  |  |
| Endpoint control |  |  |  | |  | | |  | |  |  |  |
| **Suspension** |  |  |  | |  | | |  | |  |  |  |
| Putting on socket and harness |  |  |  | |  | | |  | |  |  |  |
| Comfort of socket |  |  |  | |  | | |  | |  |  |  |
| Harnessing system |  |  |  | |  | | |  | |  |  |  |
| Dynamic straps |  |  |  | |  | | |  | |  |  |  |
| Stability of socket |  |  |  | |  | | |  | |  |  |  |
| **Tactor** |  |  |  | |  | | |  | |  |  |  |
| Vibration sensors pressure |  |  |  | |  | | |  | |  |  |  |
| Vibration sensors mode  change |  |  |  | |  | | |  | |  |  |  |
| Vibration sensors grip change |  |  |  | |  | | |  | |  |  |  |
| **Other Controls** |  |  |  | |  | | |  | |  |  |  |
| Other controls |  |  |  | |  | | |  | |  |  |  |
| Inflatable bladders |  |  |  | |  | | |  | |  |  |  |
| **Misc Individual items** |  |  |  | |  | | |  | |  |  |  |
| VRE software |  |  |  | |  | | |  | |  |  |  |
| Taking off socket and harness |  |  |  | |  | | |  | |  |  |  |
| Appearance of shoulder |  |  |  | |  | | |  | |  |  |  |
| Dynamic socket controller |  |  |  | |  | | |  | |  |  |  |
| Level of waterproofing |  |  |  | |  | | |  | |  |  |  |

**S2 Table. DEKA Arm Satisfaction scale**
